# Secondary Metabolic Profile as a Tool for Distinction and Characterization of Cultivars of Black Pepper (*Piper nigrum* L.) Cultivated in Pará State, Brazil

**DOI:** 10.3390/ijms22020890

**Published:** 2021-01-17

**Authors:** Luccas M. Barata, Eloísa H. Andrade, Alessandra R. Ramos, Oriel F. de Lemos, William N. Setzer, Kendall G. Byler, José Guilherme S. Maia, Joyce Kelly R. da Silva

**Affiliations:** 1 Programa de Pós-Graduação em Biotecnologia, Universidade Federal do Pará, Belém, PA 66075-110, Brazil; luccas.miranda2@gmail.com; 2 Coordenação de Botânica, Museu Paraense Emílio Goeldi, Belém, PA 66077-830, Brazil; eloisa@museu-goeldi.br; 3 Instituto de Estudos em Saúde e Biológicas, Universidade Federal do Sul e Sudeste do Pará, Marabá, PA 68507-590, Brazil; rezende@unifesspa.edu.br; 4 Centro de Pesquisa Agroflorestal da Amazônia Oriental, Empresa Brasileira de Pesquisa Agropecuária (EMBRAPA), Belém, PA 66095-100, Brazil; oriel.lemos@embrapa.br; 5 Aromatic Plant Research Center, 230 N 1200 E, Suite 100, Lehi, UT 84043, USA; 6 Department of Chemistry, University of Alabama in Huntsville, Huntsville, AL 35899, USA; 7 Department of Biological Sciences, University of Alabama in Huntsville, Huntsville, AL 35899, USA; kgb0011@uah.edu; 8 Programa de Pós-Graduação em Química, Universidade Federal do Maranhão, São Luís, MA 65080-805, Brazil; gmaia@ufpa.br

**Keywords:** sesquiterpenes, monoterpenes, phenolic compounds, carotenoids, PAL activity, molecular docking

## Abstract

This study evaluated the chemical compositions of the leaves and fruits of eight black pepper cultivars cultivated in Pará State (Amazon, Brazil). Hydrodistillation and gas chromatography–mass spectrometry were employed to extract and analyze the volatile compounds, respectively. Sesquiterpene hydrocarbons were predominant (58.5–90.9%) in the cultivars “Cingapura”, “Equador”, “Guajarina”, “Iaçará”, and “Kottanadan”, and “Bragantina”, “Clonada”, and “Uthirankota” displayed oxygenated sesquiterpenoids (50.6–75.0%). The multivariate statistical analysis applied using volatile composition grouped the samples into four groups: γ-Elemene, curzerene, and δ-elemene (“Equador”/“Guajarina”, I); δ-elemene (“Iaçará”/“Kottanadan”/“Cingapura”, II); elemol (“Clonada”/“Uthirankota”, III) and α-muurolol, bicyclogermacrene, and cubebol (“Bragantina”, IV). The major compounds in all fruit samples were monoterpene hydrocarbons such as α-pinene, β-pinene, and limonene. Among the cultivar leaves, phenolics content (44.75–140.53 mg GAE·g^−1^ FW), the enzymatic activity of phenylalanine-ammonia lyase (20.19–57.22 µU·mL^−1^), and carotenoids (0.21–2.31 µg·mL^−1^) displayed significant variations. Due to black pepper’s susceptibility to *Fusarium* infection, a molecular docking analysis was carried out on *Fusarium* protein targets using each cultivar’s volatile components. *F. oxysporum* endoglucanase was identified as the preferential protein target of the compounds. These results can be used to identify chemical markers related to the susceptibility degree of black pepper cultivars to plant diseases prevalent in Pará State.

## 1. Introduction

The genus *Piper* is the largest from the Piperaceae, with about 3000 species identified, and is widely distributed in tropical and subtropical regions of the planet, and in Brazil, there are around 44 varieties reported, distributed among 292 species [1,2]. The most representative species of the genus is *Piper nigrum* L. (black pepper), which is considered the “King of Spices” and is commonly used as a condiment around the world, but is also applied in traditional medicine in several countries, mainly in Asia, Africa, and South America [3,4]. Black pepper is an aromatic plant usually known for the application of its flavoring agents in the reduction of muscle pain, feverish conditions, diarrhea, gastric conditions and cholesterol, in addition to acting as an anti-inflammatory, antioxidant, antimicrobial, and repellent agent [5,6,7,8].

*Piper* species are distinguished by the presence of nodes in the shoots, inflorescence spikes, and their aromatic smell [9]. Black pepper is a perennial, woody vine that easily grows in shaded places, with indeterminate growth, but some studies report that some plantations limit their height up to 9 m. If the plant touches the ground, the root can come out of the leaf nodes [10]. Cultivars of black pepper present differences in leaf shape, stem, flowers, as well as the fruit size and thickness [11,12,13]. The species presents several cultivars identified in the literature, with dozens of these cultivars explored in the main production areas of Brazil, such as “Apra”, “Bragantina”, “Cingapura”, “Clonada”, “Equador”, “Guajarina”, “Iaçará”, “Kottanadan”, “Kuthiravally”, and “Uthirankota”, among others [13,14,15,16,17].

India, Brazil, Indonesia, Malaysia, Vietnam, China, and Sri Lanka are the largest producers of black pepper [18,19,20]. In Brazil, their economic value started increasing at the end of World War II [21]. Since then, Brazil became one of the largest producers of black pepper in the world. In 2018, the national production was about 101,000 tons, and the State of Pará, which historically was the most significant national producer, appeared as the second largest producer, with more than 33,000 tons (33% of national production). Occasionally, the State of Espírito Santo was the largest national producer of the year [22]. However, the fluctuations in annual production and product availability, and the occurrence of plant diseases, such as fusariosis (*Fusarium solani* f. sp. *piperis*) and yellow wilt (*Fusarium oxysporum*), have resulted in price instability in the international market of this condiment [21,23,24].

Secondary metabolites are defined as products of metabolism that are non-essential for the survival of a plant, but they play an important role in the interaction with the environment in which the plant is inserted, including protection against phytopathogenic microorganisms [25,26]. They are distributed in different parts of the plant (leaves, roots, fruits, seeds, or flowers), and can be obtained from extracts or in their essential oils (EOs), which have several properties, such as fungicide, bactericide and insecticide, in addition to antioxidant activity [12,27,28].

Some secondary metabolites of black pepper are used to fight infections caused by microorganisms, such as viruses and fungi, that are called phytoalexins [29,30]. Studies report that its fruit extracts have presented high bactericidal activity against *Escherichia coli*, *Staphylococcus aureus*, *Salmonella typhi*, and *Bacillus subtilis* [31]. Additionally, the EO of *P. nigrum* exhibited potential usability as a natural antioxidant and food supplement, and presents a pharmacological potential [32].

The metabolomic analysis of plants against these stimuli can help in the identification of secondary metabolites involved in different plant physiological processes, and can be used as biomarkers for the discrimination and characterization of the species [33,34,35,36]. In the literature, several studies show the effectiveness of plant extracts against different pathogens. However, more detailed analysis is required to understand compounds that are directly responsible for the antimicrobial activity and their protein–ligand interaction mechanisms [37]. Thus, molecular docking analysis has become an important tool to evaluate small-molecule ligands’ potential interactions with protein targets. The results can lead to the elucidation of biologically active agents’ mechanisms from plant sources that can assist in natural-product drug discovery against different pathogens [38]. 

The soil-borne pathogens *Fusarium solani* f.sp. *piperis* and *F. oxysporum* are the most notably responsible for the fungal diseases in black pepper that negatively interfere in its fruit production and the plant quality [39,40]. Moreover, they cause significant yield loss due to the severe damage, since they do not present apparent symptoms in the initial phase of infection [41]. Strategies of control have not been totality efficient in treating and preventing black pepper diseases. In this sense, the metabolomic analysis of black pepper cultivars combined with molecular docking studies is presented in this study. The results can be employed as an alternative in target prediction for developing new natural fungicidal agents based on their mechanisms of protein–ligand interactions. 

Therefore, this study aims to identify the secondary metabolic profile in the *P. nigrum* cultivars cultivated in the Pará State (Brazil), and to evaluate the potential volatile phytoalexins by molecular docking analysis on *Fusarium* protein targets. The discrimination and characterization of the cultivars in chemical groups can give more insight into plant–pathogen interactions, so as to develop new strategies in plant disease control.

## 2. Results and Discussion

### 2.1. Evaluation of Production and Identification of Secondary Metabolites from Cultivars of Black Pepper

#### 2.1.1. Volatile Compounds in the Leaves of Black Pepper

The chemical analysis by gas chromatography–mass spectrometry (GC-MS) resulted in the identification of 114 components (see Supplementary Material, Table S1), representing an average of 98.7% of the total composition. The predominance of sesquiterpene hydrocarbons was observed in the cultivars “Cingapura” (58.48 ± 0.94%), “Equador” (59.35 ± 5.45%), “Guajarina” (71.81 ± 4.47%), “Iaçará” (90.90 ± 1.03%), and “Kottanadan” (74.63 ± 2.38%). On the other hand, the cultivars “Bragantina” (50.55 ± 10.35%), “Clonada” (60.90 ± 3.58%), and “Uthirankota” (75.01 ± 7.89%) presented oxygenated sesquiterpenoids as the majority. The high variability of chemical composition among the cultivars can be attributed to environmental factors such as growth and development, and whether biotic or abiotic, which also directly influence the intensity of the aroma [42].

**Figure 1:**
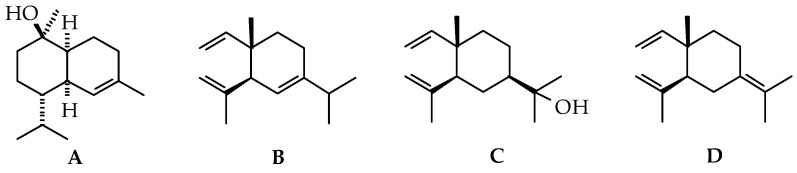
The main volatile compounds found in the leaves of black pepper cultivars. (**A**) α-muurolol; (**B**) δ-elemene; (**C**) elemol; (**D**) γ-elemene.

The “Bragantina” cultivar showed a predominance of oxygenated sesquiterpenoids (50.55 ± 10.35%), followed by sesquiterpenoid hydrocarbons (34.62 ± 12.97%). The major components were α-muurolol (20.63 ± 2.81%) (Figure 1A), bicyclogermacrene (7.55 ± 4.95%), cubebol (6.49 ± 2.11%) and δ-cadinene (6.04 ± 1.77%). In another study, the major compounds identified in the cultivar “Bragantina” were cubenol (14.55–21.53%), bicyclogermacrene (6.36–8.16%), and β-selinene (4.10–6.21%) [43]. The main component of “Bragantina” cultivar, α-muurolol, can also be found in other *Piper* species, such as *P. amalago* (5.0–9.3%) [44], *P. caldense* (9.0%) [45], *P. cernuum* Vell. (5.8%) [46], *P. manausense* (7.6%) [47], and *P.* sp. aff. *aereum* (5.8%) [48].

In the “Cingapura” cultivar, sesquiterpene hydrocarbons (58.48 ± 0.94%) were predominant, followed by oxygenated sesquiterpenoids (20.36 ± 0.80%). The major components identified were δ-elemene (37.17 ± 1.90%) (Figure 1B), linalool (6.75 ± 0.37%), spathulenol (5.85% ± 0.48%), (*E*)-β-caryophyllene (5.72 ± 0.93%), and muurola-4,10(14)-dien-1-β-ol (4.83 ± 0.64%). “Iaçará” and “Kottanadan” cultivars displayed similar chemical profiles with quantitative differences. The main compounds were δ-elemene (50.89 ± 1.64% and 32.1% ± 7.44%), β-elemene (9.92 ± 0.58% and 6.10 ± 1.32%), viridiflorene (8.87 ± 0.84% and 12.42 ± 2.28%), and β-selinene (8.17 ± 0.57% and 7.54 ± 2.59%) for the “Iaçará” and “Kottanadan” cultivars, respectively. In black pepper, δ-elemene (53.3%) was previously reported as the main component in the leaves of “Cingapura” cultivar. The other main constituents identified in the cultivar were (*E*)-β-caryophyllene (4.7%), and muurola-4,10(14)-dien-1β-ol (4.9%) [49]. Other *Piper* species that present δ-elemene as one of the main constituents are *P. aequale* (19.0%) [50], *P. amalago* (6.82%) [51], *P. aleyreanum* (8.2%) [52], *P. artanthe* (11.69%) [53], *P. dilatatum* (7.6%) [54], and *P. fimbriulatum* (9.4%) [48]. The major compounds in the “Clonada” and “Uthirankota” cultivars were oxygenated sesquiterpenoids (60.90 ± 3.58% and 75.01 ± 7.89%, respectively). Elemol (40.55 ± 4.87%) (Figure 1C) and α-bisabolol (17.97 ± 0.97%) were identified as the main components. In black pepper, α-bisabolol (32.3%) and elemol (11.4%) were identified in the leaves of cultivar “Bragantina” [55]. Moreover, elemol is one of the main constituents of *P. cernuum* (5.89–12.0%) [56,57,58], *P. hispidum* (7.6%) [59], and *P. marginatum* (5.9–9.7%) [60,61]. For α-bisabolol, *P. madeiranum* (7.1%) [62] and *P. molicomum* (9.9%) [63] showed high contents of this oxygenated sesquiterpenoid.

“Equador” and “Guajarina” cultivars presented the same chemical profile, only with quantitative differences. The main compounds were γ-elemene (38.33 ± 3.80% and 44.36 ± 4.06%) (Figure 1D), curzerene (23.21 ± 4.96% and 23.39 ± 4.45%), and δ-elemene (6.93 ± 0.77% and 13.84 ± 0.80%), respectively. Several *Piper* species present γ-elemene as one of the main components in their EOs, such as *P. gaudichaudianum* Kunth (5.4%) [41], *P. marginatum* (3.75%) [64], *P. nemorense* (6.8%) [48], and *P. vicosanum* (14.16%) [65].

Curzerene was widely found in “Equador” (23.21 ± 4.96%) and “Guajarina” (23.39 ± 4.45%) cultivars, and is considered a derivative of furanodiene, which is produced from a reversible thermal rearrangement known as the Cope rearrangement [66]. It is a type of reaction characterized by the substitution of alkenes when the compound is found at high temperatures [67], which suggests that curzerene may have been produced during the essential oil extraction as well as during the gas chromatographic analysis [68]. 

In *Piper* species, this oxygenated sesquiterpenoid can be found in *P. dilatatum* (13.8–28.7%) [54] and *P. hispidum* (4.9–12.9%) [59,69]. Another well-known species that presents curzerene as one of the main components is *Eugenia uniflora*, commonly known as “pitanga”, an arboreous plant widely distributed in Brazil and other South American countries and possesses antifungal activity against *Candida* spp., and its EO presents antiproliferative and cytotoxic effects against some cancer strains [70,71].

#### 2.1.2. Volatile Compounds in the Fruits of Black Pepper

**Figure 2:**
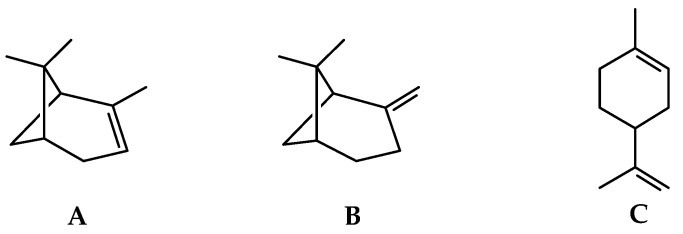
Main volatile compounds found in the fruits of all cultivars of black pepper. (**A**) α-pinene; (**B**) β-pinene; (**C**) limonene.

GC-MS analysis identified 104 components, representing 99.45% of the total composition (see Supplementary Material, Table S2). Monoterpene hydrocarbons (average of 76.1%) were predominant in all cultivars, such as α-pinene (6.91–10.67%), β-pinene (22.61–35.05%) and limonene (21.0–31.77%). The presence of α-pinene, β-pinene, and limonene (Figure 2) in the chemical composition can be found in most cultivars of black pepper [72]. The literature widely reports the influence of terpene levels on the aroma and flavor of black pepper fruits. Despite different geographical regions, the pungency and flavor of black pepper fruits are related to these compounds, with variations only in their percentages among the cultivars [72,73].

The EO chemical composition of black pepper cultivars can present quantitative and qualitative differences according to their geographic occurrence. For example, in cultivars from India (“Indigenous” and “Kerala”), quantitative variations in amounts of α-pinene (7.3–16.7%), β-pinene (13.2–13.6%) and limonene (15.2–16.2%), δ-3-carene (9.2–32.6%), α-phellandrene (2.9–8.9%), and (*E*)-β-caryophyllene (10.7–18.4%) were observed [74]. Fruits from “Sreekara”, “Kuching”, and “Vellanamban” cultivars, also found in India, present α-pinene (1.7–5.5%), β-pinene (3.9–11.2%) and limonene (8.3 22.1%) in their EO composition. However, they also display other majorities, such as (*E*)-β-caryophyllene (16.8–39.1%), sabinene (4.3–18.8%), myrcene (2.0 9.6%), α-phellandrene (3.0–7.7%, only in “Sreekara” and “Kuching” cultivars), δ-3-carene (5.1–11.1%, only in “Sreekara” and “Vellanamban” cultivars), α-cubebene (4.8–6.1%, only in the “Vellanamban” cultivar), and even the oxygenated sesquiterpene elemol (4.2–10.5, only in the “Vellanamban” cultivar) [75]. In Cameroon, the EO of black pepper presented δ-3-carene (18.5%), limonene (14.7%), (*E*)-β-caryophyllene (12.8%), sabinene (11.2%), α-pinene (5.6%), and β-pinene (6.7%), while the EO of black pepper from S. Tomé e Príncipe displayed limonene (18.8%), (*E*)-β-caryophyllene (15.4%), sabinene (16.5%) and β-pinene (10.7%) as majorities [76,77].

#### 2.1.3. Hierarchical Cluster Analysis (HCA) 

**Figure 3:**
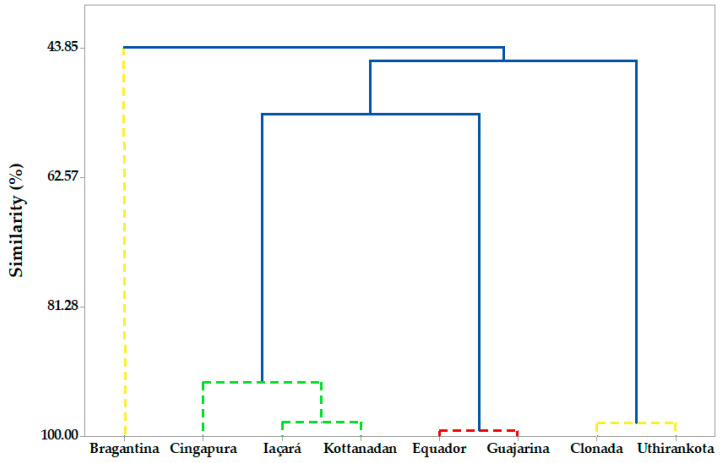
Hierarchical cluster analysis (HCA) analysis from volatile concentrates of black pepper. Different colors in the groups indicate the potential degrees of susceptibility of the cultivars to *Fusarium oxysporum* (yellow wilt disease). 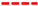 Susceptible; 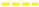 Medium tolerance; 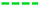 Tolerant.

Multivariate analysis based on the leaf volatiles classified the cultivars into four groups (Figure 3). Group I exhibited a similarity level of 99.2% between the cultivars “Equador” and “Guajarina”, and presented high levels of γ-elemene (38.33–44.36%), curzerene (23.21–23.39%) and δ-elemene (6.83–13.84%). This group displayed a similarity of 53.6% with group II. High concentrations of δ-elemene (30.11–50.89%) characterized the group II with a similarity level of 92.3% among the cultivars “Iaçará”, “Kottanadan”, and “Cingapura”.

In addition, cultivars “Iaçará” and “Kottanadan” presented significant amounts of viridiflorene (8.87–12.42%), β-selinene (7.54–8.17%) and β-elemene (6.10–9.92%). In contrast, the cultivar “Cingapura” exhibited spathulenol (5.85%) and (*E*)-β-caryophyllene (5.72%) as the majority. This group showed a similarity of 45.9% with group III. The cultivars “Clonada” and “Uthirankota” were grouped in Group III with a similarity level of 98.1% and high content of elemol (40.55–49.78%), and presented a similarity of 43.9% with group IV. Finally, group IV separated only the “Bragantina” cultivar from the others, due to its composition rich in α-muurolol (20.63%), bicyclogermacrene (7.55%), and cubebol (6.49%). Regarding volatiles from the fruits, two clusters were identified with a high similarity between them (~93.5%), displaying no significant variation among the cultivars.

The use of metabolic profiling as a tool for the discrimination of cultivars of several crops has been reported in the literature [78,79]. Principal component analysis (PCA) and HCA techniques classified the EOs from five varieties of *P. betle* into three groups. Group I, which englobes “Misti” and “BARI Paan 3” varieties, presented chavicol (3.97–11.95%), (*E*)-β-caryophyllene (4.24–4.69%), and valencene (3.02–3.11%). Group II was composed only of the variety “Bangla” due to the presence of eugenyl acetate (1.50%) and α-humulene (1.35%) in its composition. The varieties “Sanchi” and “Khasia” were separated in Group III, which was characterized by γ-muurolene (1.74–4.49%) and (*E*)-β-caryophyllene (1.74–4.93%) in their compositions [80].

PCA analysis of volatile profiles was able to distinguish fourteen *Citrus* varieties as tolerant and moderately tolerant from those susceptible to *Candidatus Liberibacter asiaticus* (CLas). Tolerant and moderately tolerant cultivars showed higher amounts of volatiles than susceptible varieties. These compounds include aldehydes (undecanal, neral, geranial, and citronellal) and some monoterpenes such as linalool (0.1–68.3%), d-limonene (0.3–55.3%), myrcene (0.1–8.5%), α- (0.2–3.1%) and β-phellandrene (0.1–11.4%) [81].

Previous studies reported the different degrees of the susceptibility of black pepper cultivars to *F. oxysporum* (yellow wilt) [24,82,83]. Our results indicate a possible correlation between resistance to yellow wilt and the volatile composition of the leaves. “Iaçará”, “Kottanadan”, and “Cingapura” (group II) are highly resistant, while “Bragantina” (group IV) and “Uthirankota” (group III) present medium resistance, and “Guajarina” (group I) are more susceptible. Consequently, the cultivar “Equador” may be classified as susceptible due to its high similarity with the cultivar “Guajarina” (99.2%). Furthermore, the variety “Clonada” may have a medium resistance due to its proximity to the cultivar “Uthirankota” (98.1%) (see Figure 3). 

In comparison to the fruits, the volatile profiles of leaves functioned as suitable biomarkers for the discrimination of cultivars, and their resistance or susceptibility to yellow wilt. However, further experiments with *Fusarium* inoculation given to each variety are required to confirm this relationship. Moreover, an antimicrobial assay with the main compounds is recommended for understanding the role of specific compounds in each cultivar’s plant defense mechanism.

#### 2.1.4. Total Phenolics Determination

**Figure 4:**
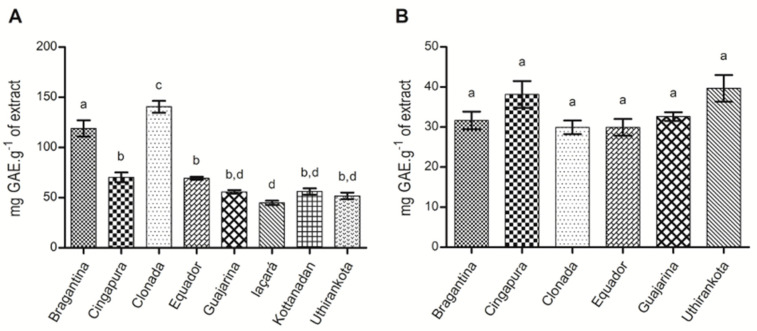
Total phenolic compounds produced in black pepper cultivars. (**A**) Leaves; (**B**) fruits. ^a–d^ Different letters represent a statistically significant difference by Tukey’s test (*p* < 0.05).

The total phenolic contents in the *P. nigrum* extracts showed great variation in the leaves (44.75 ± 1.60 to 140.53 ± 14.01 mg GAE·g^−1^) (Figure 4A). However, in the fruits we noticed only small changes in the “Cingapura” and “Uthirankota” cultivars (Figure 4B). According to the literature, these variations can be occasioned by several factors, biotic and abiotic, such as plant defense, senescence, plant development, and interactions with the environment in which they are inserted [84].

The levels of total phenolics were higher in the leaves in comparison to the fruits. These differences can be related to the harvesting and processing steps involved in sun-drying, which alter the interaction between the enzymes related to phenolic production in the black pepper fruits [85]. In addition, the content of phenolic compounds can be considered a useful parameter for determining plants’ biological functions [86]. Six black pepper varieties (“Sreekara”, “Subhakara”, “IISR Malabar Excel”, “Panniyur-1”, “Panchami” and “IISR Thevam”), and other *Piper* species (*P. chaba* Hunter, *P. longum* L., and *P. colubrinum* Link.) were extracted with *n*-hexane, chloroform, methanol, and water. Methanol extracts from “IISR Malabar Excel” and “Panchami” varieties, and a chloroform extract of *P. colubrinum* displayed higher phenolic contents, with values of 50.85, 38.74, and 100.6 mg GAE·g^−1^ of the extract [87]. Therefore, our findings suggest that black pepper cultivars cultivated in the Pará State, Brazil have high phenolic contents compared to other cultivars from other regions of the world, and the extracts may present several biological activities.

Plants that are under biotic or abiotic stresses can exhibit changes in their phenolic contents in the early stages of development [88]. Black pepper, after infection with *F. solani* f. sp. *piperis*, showed a significant variation in total phenolics in the leaves and roots from the “Cingapura” cultivar (tolerant to *Fusarium*), which can be attributed to the response to the infection in the early stages, since the first symptoms were noted after two weeks of inoculation [49]. Moreover, the association of the “Bragantina” cultivar of black pepper with arbuscular mycorrhizal fungi (AMF) induced an increase in phenolic content, which was correlated to changes in enzymatic activity linked to metabolic pathways related to plant defense. These findings suggest that AMF can induce a better resistance to pathogens [43].

#### 2.1.5. Total Carotenoids

**Figure 5:**
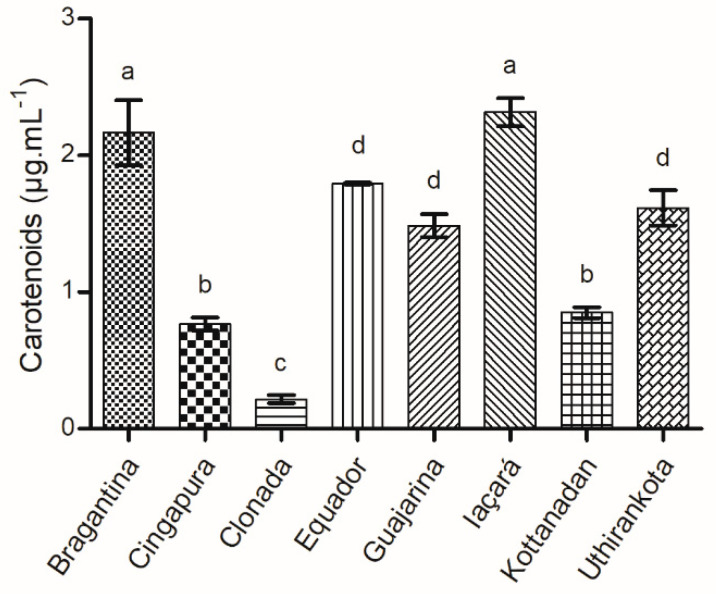
Total carotenoids produced in the leaves of black pepper cultivars. ^a–d^ Different letters represent a statistically significant difference by Tukey’s test (*p* < 0.05).

The concentration of total carotenoids revealed a great variation among the cultivars (0.21 ± 0.03 to 2.31 ± 0.10 µg·mL^−1^) (Figure 5). The literature reports a synergistic effect of carotenoids of great importance for the biological properties of plant extracts, which is linked to the production of different classes of compounds such as phenylpropanoids, terpenes, and alkaloids [89].

In plants, the carotenoids are involved in different areas, such as antioxidants, accessory pigments that absorb light, and molecules that act as attractants for pollinators and seed dispersers, in addition to the plant response to environmental stresses [90]. Thus, it is possible to measure the impact of the environment on plant metabolism. An increase in carotenoid concentration was observed in the black pepper cultivar “Panniyur 1”, which is tolerant to drought stress, suggesting a relationship between the production of these compounds and the mechanisms of tolerance to stress [91]. Another study showed the effect of natural growth promoters on the physiological and biochemical development of the cultivar “Panniyur 1” of black pepper, and displayed an increase of 159% in total carotenoids compared with the control group. Therefore, species that have access to a greater amount of the nutrients essential to plant growth also tend to have a higher concentration of carotenoids [92].

### 2.2. Comparison of Enzymatic Activity In Vitro in the Leaves of Black Pepper Cultivars

#### Phenylalanine Ammonia-Lyase (PAL) Activity

**Figure 6:**
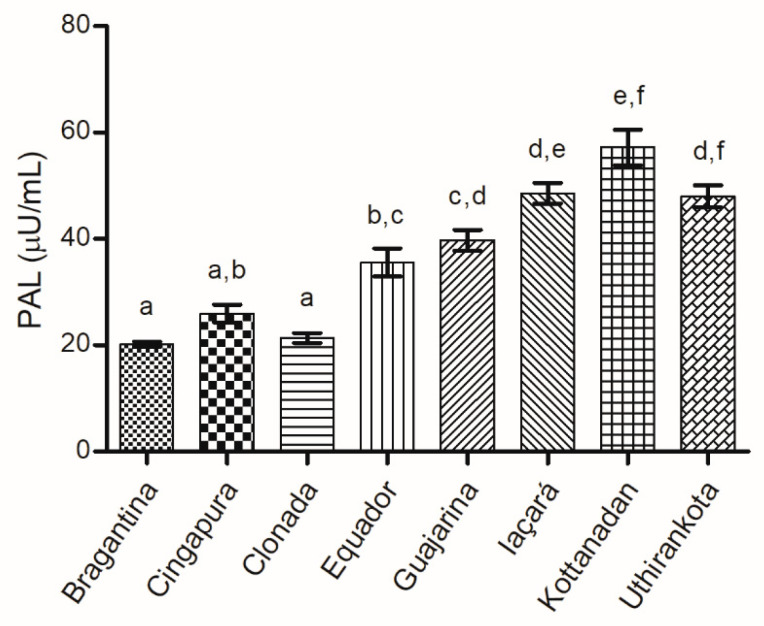
Enzymatic activity of PAL in the leaves of black pepper cultivars. ^a–f^ Different letters represent a statistically significant difference by Tukey’s test (*p* < 0.05).

*P. nigrum* cultivars exhibited a considerable variation in PAL enzymatic activity, ranging from 20.19 to 57.22 µU·mL^−1^ (Figure 6). PAL is a key enzyme in the biosynthetic pathway of phenylpropanoids, which displays an essential role in the production of metabolites related to plant defense, such as flavonoids, alkaloids, lignans, lignins, and coumarins [93].

Alkaloids are the main class produced by the shikimic acid pathway present in black pepper, especially the piperamides [94]. These metabolites are present in the fruits and seeds of black pepper, and have wide pharmacological applicability, such as in antimetastatic, antidepressant, hepatoprotective and antitumor activities, and they also have neuroprotective effects [95,96]. Alkaloid concentrations and their biological properties are directly related to PAL activity in the plant [97,98].

An increase in PAL activity can result from a sensitive response that occurs in plants when they are infected by pathogens or during an injury [99,100]. Significant changes in PAL activity were observed in the cultivars “Cingapura” and “Trau” of black pepper, two days after infection with *Phytophthora capsici*. The results showed that PAL causes an immediate response in the production of defense metabolites when the plant is under stress [101]. After 48 h of co-inoculation with two strains of rhizobacteria (*Bacillus subtilis* and *Pseudomonas fluorescens*) and two strains of endophytic fungi (*Trichoderma viride* and *Trichoderma asperellum*), an increase in PAL activity induced the systemic resistance of black pepper against *P. capsici* [102]. Therefore, the quantification of PAL activity can be considered an alternative in the evaluation of plant resistance against infection by pathogens.

### 2.3. Homology Modeling and Molecular Docking to Fusarium

In an attempt to correlate volatile phytoalexins with *Fusarium* resistance, we have carried out a molecular docking analysis of the major *P. nigrum* volatile components with *Fusarium* protein targets that were available from the Protein Data Bank (PDB): *F. oxysporum* cutinase (PDB 5AJH), *F. solani* cutinase (PDB 1AGY, 1XZL, and 1XZM), *F. oxysporum* endoglucanase (PDB 4OVW), *F. oxysporum* feruloyl esterase (PDB 6FAT), and *F. oxysporum* xylanase (PDB 5JRM). In addition, *F. solani* ornithine decarboxylase was prepared by homology modeling using murine ornithine decarboxylase (PDB 7ODC) as a template, *F. oxysporum* glucosamine-fructose-6-phosphate aminotransferase was prepared from human glutamine fructose-6-phosphate amidotransferase (PDB 6R4E) as a template, *F. odoratissimum* β-glucosidase was prepared using *Neurospora crassa* β-glucosidase (PDB 5NBS) as the template, *F. oxysporum* guanine nucleotide-binding protein subunit β was prepared using human guanine nucleotide-binding protein subunit α1 (PDB 6CMO) as the template, and *F. vanettenii* thiamine thiazole synthase was prepared using *N. crassa* thiazole synthase (PDB 3JSK) as the template. Docking energies for the essential oil ligands and 10 *Fusarium* target proteins are listed in Supplementary Material, Table S3.

**Figure 7:**
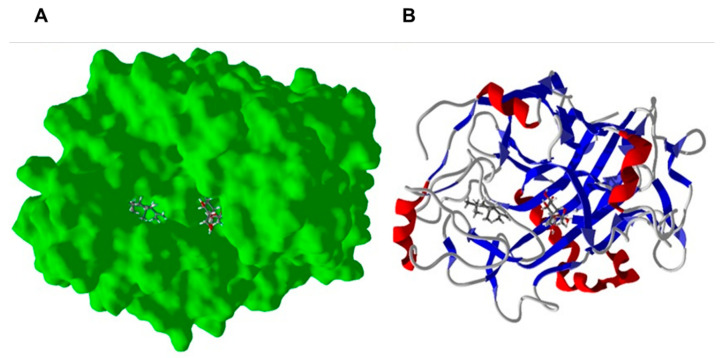
Lowest-energy docked pose of δ-elemene (left stick figure) with *Fusarium oxysporum* endoglucanase (Protein Data Bank, PDB 4OVW). The co-crystallized ligand (right stick figure) is 4-(β-D-glucopyranosyloxy)-2,2-dihydroxybutyl propanoate. (**A**): Solid protein structure. (**B**): Ribbon structure.

The major components found in the *Fusarium*-resistant *P. nigrum* cultivars (“Cingapura”, “Iaçará”, and “Kottanadan”) were δ-elemene (abundant in all three cultivars), viridiflorene, β-selinene, β-elemene (abundant in “Iaçará” and “Kottanadan”), spathulenol and (*E*)-β-caryophyllene (abundant in “Cingapura”). δ-Elemene, viridiflorene, β-elemene, and (*E*)-β-caryophyllene, all showed selective docking (most exothermic docking energies) to *F. oxysporum* endoglucanase. Furthermore, *F. oxysporum* endoglucanase had the lowest docking average energy for the 40 essential oil components, and was the most targeted protein with 13 ligands showing preferential docking to this protein. Endoglucanase is one of the enzymes responsible for breaking down cellulose by hydrolyzing the β-1,4-glycosidic bonds in the cellulose polymer [103]. Thus, the inhibition of this protein target would protect the plant from cellulose degradation by *Fusarium*. Interestingly, the essential oil components all dock into a hydrophobic pocket removed from the site of the co-crystallized ligand (4-(β-*D*-glucopyranosyloxy)-2,2-dihydroxybutyl propanoate) but still in the active canyon of the enzyme (Figure 7).

## 3. Materials and Methods

### 3.1. Plant Material

The leaves and fruits of “Bragantina”, “Cingapura”, “Clonada”, “Equador”, “Guajarina”, “Iaçará”, “Kottanadan”, and “Uthirankota” cultivars of *Piper nigrum* L. (Piperaceae) were provided by EMBRAPA Amazônia Oriental (Brazilian Agricultural Research Corporation). The plants were acclimatized and maintained in a greenhouse with a daily watering regime. Adult leaves were collected during the fruiting stage and stored at −20 °C for further experimentation. For the fruits, the collection time varied from July to October, according to its ripeness for each cultivar. After harvesting, the fruits were sun-dried for about three days until the humidity was around 13%, and preserved at room temperature for further experimentation.

### 3.2. Identification of Secondary Metabolites from Cultivars of Black Pepper

#### 3.2.1. Extraction and Analysis of Volatile Organic Compounds (VOCs)

Leaves and fruits were extracted by the simultaneous distillation–extraction process using a Likens–Nickerson apparatus to obtain the volatile concentrate. The solvent of the extraction was *n*-pentane (3 mL) for two hours. After extraction, an aliquot (1.0 μL) of the organic phase was injected in a gas chromatography coupled with mass spectrometry (GC-MS) apparatus. The analysis was performed in GC-MS equipment (Shimadzu QP2010 ultra, Shimadzu Corporation, Tokyo, Japan) according to the following settings: Rtx-5MS (30 m × 0.25 mm × 0.25 µm film thickness), silica capillary column (Restek Corporation, Bellefonte, PA, USA); programmed temperature, 60–240 °C (3 °C/min); injector temperature, 250 °C; carrier gas, helium with linear velocity of 32 cm/s (measured at 100 °C); injection type, split (1.0 µL); split flow was adjusted to yield a 20:1 ratio; septum sweep was a constant 10 mL/min; EIMS, electron energy, 70 eV; temperature of the ion source and connection parts, 200 °C. A homologous series of *n*-alkanes (C8–C32, Sigma-Aldrich, Milwaukee, WI, USA) was applied to calculate the retention index (RI) [104], which were used in conjunction with the mass spectra to identify the compounds found in the libraries of Adams and FFNSC2 [105,106]. The compound percentages are based on peak integrations without standardization.

#### 3.2.2. Extracts Preparation

Samples (2.0 g) of leaves and fruits of *P. nigrum* were extracted by percolation (96 h) with 100 mL of methanol. Every 48 h, the samples were placed on ultrasound for 10 min. At the end of 96 h, the solvent was evaporated. The extracts were used for Folin–Ciocalteu total phenolic and total carotenoids determination [107,108,109].

#### 3.2.3. Total Phenolics Determination

The extracts were solubilized in methanol (20 mg·mL^−1^), followed by dilution in distilled water. Then, an aliquot of 500 μL of extract was added to 250 μL of Folin–Ciocalteu reagent (1 N) and 1250 μL of sodium carbonate (75 g·L^−1^). The reaction was kept in the dark and after 30 min, the absorbance of the mixture was read at 760 nm using a UV-visible spectrophotometer (Amersham Biosciences, Little Chalfont, UK). The experimental calibration curve was prepared using gallic acid (Sigma Aldrich, St. Louis, MO, USA) at concentrations of 0.5 to 10.0 mg·L^−1^, and the content of total phenolics was expressed as gallic acid equivalents (GAE) in milligrams per gram of extract (mg GAE·g^−1^).

#### 3.2.4. Total Carotenoids

An aliquot of each extract was prepared in ethanol at the concentration of 1 mg·mL^−1^. The absorbance (*Abs*) of the samples was read at 470, 648, and 664 nm using a UV-visible spectrophotometer (Amersham Biosciences, Little Chalfont, UK). Ethanol was used as a blank. The pigment contents (chlorophyll *a—Chl_A_*, chlorophyll *b—Chl_B_*, and total carotenoids—*Car_total_*) were calculated using Equations (1)*–*(3). The carotenoid content was expressed in µg of extract per mL of ethanol (µg·mL^−1^).

(1) $${Chl}_{A}=\left(13.36*Abs_{664}\right)-\left(5.19*Abs_{648}\right)\;$$

(2) $${Chl}_{B}=\left(27.43*Abs_{648}\right)-\left(8.12*Abs_{664}\right)\;$$

(3) $$Car_{total}=\frac{\left(\left(1000*{\mathrm{Abs}}_{470}\right)-\left(2.13*{\mathrm{Chl}}_{A}\right)-\left(97.64*{\mathrm{Chl}}_{B}\right)\right)}{209}\;$$

### 3.3. Determination of In Vitro Enzymatic Activity from Leaves of Black Pepper Cultivars

#### Phenylalanine Ammonia-Lyase (PAL) Activity

Frozen fresh leaves of each cultivar were pulverized in liquid nitrogen. Each sample (100 mg) was homogenized in sodium borate buffer (0.3 mM, pH 8.8), 1 mM ethylenediaminetetraacetic acid (EDTA) (Sigma Aldrich, St. Louis, MO, USA), 1 mM dithiothreitol (DTT) (Sigma Aldrich, St. Louis, MO, USA), and 5% polyvinylpolypyrrolidone (PVP) (Sigma Aldrich, St. Louis, MO, USA), totalizing 1 mL of extraction buffer. The samples were centrifuged at 12,000× *g* (15 min at 4 °C), and then an aliquot of supernatant (0.5 mL) was mixed with 1.0 mL of reaction buffer composed of sodium borate buffer (0.3 mM, pH 8.8), and 0.03 mM L-phenylalanine (Sigma Aldrich, St. Louis, MO, USA). After 15 min at room temperature, the absorbance was measured at 290 nm using a UV-visible spectrophotometer (Amersham Biosciences, Little Chalfont, UK). The specific molar extinction coefficient of (*E*)-cinnamic acid (9630 mol·L^−1^·cm^−1^) was used to determine the PAL activity based on its formation from the substrate, L-phenylalanine [110,111].

### 3.4. Computational Methods

#### 3.4.1. Homology Modeling

Homology models for each of the *Fusarium* targets were generated using three-dimensional crystal structures from the Protein Data Bank (PDB, http://www.rcsb.org/) using FASTA sequences obtained from the National Center for Biotechnology Information (NCBI) protein database (https://www.ncbi.nlm.nih.gov/protein) (see Table 1). Protein sequences in the NCBI GenBank with high sequence similarity to structures in the PDB were identified using the PSI-BLAST utility with the BLOSUM80 scoring matrix. Structures with both high similarity to the selected sequence, in addition to a good overlap with the active sites for the protein, were selected for single-reference homology modeling. The protein sequence was then aligned to its respective structural template using the BLOSUM62 substitution matrix. As necessary, either the FASTA sequence or the reference sequence was extended with gaps. Protein structures were generated by the alignment of atomic coordinates of the peptide sequence with those of the template backbone. Side-chain orientations were carried out using the AMBER14:EHT force field with reaction field solvation [112,113,114]. The protein structure with the lowest deviation from the template backbone was selected and optimized using a constrained minimization. The sequence of each structure was aligned to its respective template and the protein backbone was created and superposed to the reference structures using the protein alignment tool in MOE 2019.01 (Chemical Computing Group, Montreal, QC, Canada).

#### 3.4.2. Molecular Docking

Molecular docking was carried out on 39 *P. nigrum* essential oil major components with 7 *Fusarium* protein targets from the Protein Data Bank (PDB, http://www.rcsb.org/): *F. oxysporum* cutinase (PDB 5AJH), *F. solani* cutinase (PDB 1AGY, 1XZL, and 1XZM), *F. oxysporum* endoglucanase (PDB 4OVW), *F. oxysporum* feruloyl esterase (PDB 6FAT), and *F. oxysporum* xylanase (PDB 5JRM). In addition, homology models of *F. solani* ornithine decarboxylase, *F. vanettenii* thiamine thiazole synthase, *F. oxysporum* glucosamine-fructose-6-phosphate aminotransferase, *F. odoratissimum* β-glucosidase, and *F. oxysporum* guanine nucleotide-binding protein subunit β, were also screened. Structures of the ligands were generated using Spartan ’18 for Windows version 1.4.4 (Wavefunction, Inc., Irvine, CA, USA). Conformational analyses and geometry optimizations were carried out using the MMFF force field [115]. The ligands were screened using Molegro Virtual Docker version 6.0.1 (Molegro ApS, Aarhus, Denmark) [116]. Solvent molecules and co-crystallized ligands were removed from the protein structure prior to docking. A binding site sphere large enough to accommodate the binding cavity was positioned on the binding site of each protein. Protonation states of the homology model residues at neutral pH were assigned. The protein structure was used as a rigid model (i.e., no relaxation of the protein structure was performed). The assignment of charges was based on standard defaults of the Molegro Virtual Docker program. The ligands were used as flexible models in both the docking and succeeding optimization schemes. Various orientations of the ligands with the protein were searched and ranked based on their re-rank docking scores. For each docking run the number of iterations for the docking procedure was set to a maximum of 1500, with a maximum population size of 50, and a total of 100 runs per ligand. A threshold of 1.00 Å RMSD was set for multiple poses. The poses generated for each ligand were then sorted by the calculated re-rank docking scores.

### 3.5. Statistical Analysis

The multivariate analysis was performed using the Minitab^®^ software, version 18.1 (Minitab Inc, State College, PA, USA), using as variables the chemical components that are exhibited at least once in the composition of the cultivars and can reach above 1%. The data matrix was standardized by subtracting the mean from the individual value of each compound and then subtracting it by the standard deviation. The Euclidian distance, complete linkage, and absolute correlation coefficient distance were selected to obtain the similarity of the cultivars for the hierarchical cluster analysis (HCA). For total phenolics determination, total carotenoids, and PAL activity, in each sample, three independent tests were performed, and the results were expressed as mean ± standard deviation. The data were submitted to a normality test for validation (ANOVA), and subsequently, the Tukey’s test was applied with a 5% probability level (*p* < 0.05), using GraphPad Prism 6.0 software (GraphPad Software, Inc. San Diego, CA, USA).

## 4. Conclusions

The present study detailed the qualitative and quantitative composition of volatile concentrates from the leaves and fruits of black pepper cultivars, which revealed about 218 components (114 in the leaves and 104 in the fruits). HCA analysis allowed the chemical discrimination of the cultivars based on the biomarkers identified in the aroma of the leaves. Differences among the cultivars were also observed in the total phenolics content, PAL activity, and total carotenoids. Although molecular docking was used to evaluate the potential inhibition of *Fusarium* enzyme targets, and *Fusarium* endoglucanase was identified as a potential enzyme target, there are several other mechanisms of anti-*Fusarium* activity that may be important. Nevertheless, it was possible to correlate the metabolomic analysis with the susceptibility degree to yellow wilt of black pepper cultivars available in the State of Pará, Brazil, and the results highlight the importance of secondary metabolites in understanding the interaction of plants with different environments for the development of new biotechnologies.

## Figures and Tables

**Table 1:** Structure templates from PDB used for homology modeling for the *Fusarium* enzymes.

Target	GenBank Accession Number	PDB Entry	Sequence Identity	Expectation Value
*F. solani* ornithine decarboxylase	ABC47117.1	4ZGY	51.23%	4 × 10^−108^
*F. oxysporum* glucosamine fructose-6-phosphate aminotransferase	EWY94476	6R4E	61.11%	0.0
*F. odoratissimum* NRRL 54006 β-glucosidase	XP_031065848	5NBS	65.47%	0.0
*F. vanettenii* thiamine thiazole synthase	C7Z8P6.1	3JSK	77.4%	1 × 10^−167^
*F. oxysporum* guanine nucleotide-binding protein, beta subunit	RKL24731	6CMO	66.57%	2 × 10^−165^

## Data Availability

The data presented in this study are available within the article or supplementary material.

## Supplementary Materials

Supplementary materials can be found at https://www.mdpi.com/1422-0067/22/2/890/s1. Table S1. Volatile chemical composition of the leaves of black pepper cultivars (Mean ± standard deviation - %). Table S2. Identification of chemical composition in the fruits of black pepper cultivars (Mean ± standard deviation - %). Table S3. Docking energies (kJ/mol) for the essential oil ligands and their respective *Fusarium* target proteins.

## Abbreviations

*Abs*	Absorbance
AMF	Arbuscular mycorrhizal fungi
ANOVA	Analysis of variance
*Car_total_*	Total carotenoids
*Chl_A_*	Chlorophyll A
*Chl_B_*	Chlorophyll B
CLas	*Candidatus Liberibacter asiaticus*
DTT	Dithiothreitol
EDTA	Ethylenediaminetetraacetic acid
EIMS	Electron ionization mass spectrometry
EMBRAPA	Brazilian Agricultural Research Corporation
EO	Essential oil
GAE	Gallic acid equivalents
GC-MS	Gas chromatography–mass spectrometry
HCA	Hierarchical cluster analysis
NCBI	National Center for Biotechnology Information
PAL	Phenylalanine ammonia-lyase
PCA	Principal component analysis
PDB	Protein Data Bank
PVP	Polyvinylpolypyrrolidone
RI	Retention indices
VOC	Volatile organic compounds
