# Supplementary material for: Secondary Metabolic Profile as a Tool for Distinction and Characterization of Cultivars of Black Pepper (Piper nigrum L.) Cultivated in Pará State, Brazil
Source: Int J Mol Sci. 2021 Jan 17;22(2):890. doi: 10.3390/ijms22020890 (PMC7830865; doi:10.3390/ijms22020890)
Supplement: Supplementary file 1 [file ijms-22-00890-s001.pdf]

International Journal of *Molecular Sciences*

MDPI

Supplementary Material

Table S1. Volatile chemical composition of the leaves of black pepper cultivars (Mean ± standard deviation - %).

RI (C)	RI (L)	Compound	Bragantina	Cingapura	Clonada	Equador	Guajarina	Iaçará	Kottanadan	Uthirankota
768	762	1-Pentanol	1.62 ± 0.35	1.56 ± 0.06	0.98 ± 0.28	1.08 ± 0.76			1.51 ± 0.90	
788	788	1-Octene	3.13 ± 0.35	3.23 ± 0.27	1.70 ± 0.10	1.69 ± 1.17			2.65 ± 1.50	
819	815	(2*E*)-Octene	0.19 ± 0.27	0.44 ± 0.03						
832	833	3-Methylpentanol	1.95 ± 0.97	0.48 ± 0.44					0.13 ± 0.22	
846	846	(2*E*)-Hexenal	2.73 ± 0.45	2.61 ± 1.08			0.11 ± 0.18		0.24 ± 0.41	
921	930	Tetrahydrocitronellene		0.02 ± 0.03						
969	974	β-Pinene		0.08 ± 0.14						
992	1000	*n*-Decane		0.21 ± 0.05						
1022	1024	Limonene		0.40 ± 0.42						
1045	1044	(*E*)-β-Ocimene		0.47 ± 0.11		2.35 ± 1.87				0.21 ± 0.03
**1093**	**1095**	**Linalool**	0.90 ± 0.30	**6.75 ± 0.37**	1.96 ± 0.28	0.11 ± 0.18	0.94 ± 0.17	0.28 ± 0.04	0.48 ± 0.24	0.61 ± 0.07
1152	1148	Citronellal								0.03 ± 0.05
1255	1257	Methyl citronellate	0.37 ± 0.35	0.87 ± 0.06					0.02 ± 0.03	
1270	1274	Pregeijerene B			0.32 ± 0.01					
1293	1293	2-Undecanone	0.23 ± 0.04	0.50 ± 0.16	0.08 ± 0.11	0.02 ± 0.03	0.20 ± 0.05	0.08 ± 0.07	0.02 ± 0.03	0.21 ± 0.02
1324	1334	Linalyl propanoate							0.05 ± 0.08	
**1335**	**1335**	**δ-Elemene**	3.21 ± 0.63	**37.17 ± 1.90**	1.76 ± 0.01	**6.93 ± 0.77**	**13.84 ± 0.80**	**50.89 ± 1.64**	**32.11 ± 7.44**	0.07 ± 0.06
1345	1345	α-Cubebene	1.73 ± 0.12	0.48 ± 0.03	0.67 ± 0.10	0.04 ± 0.06	0.02 ± 0.04	1.00 ± 0.23	0.96 ± 0.47	
1371	1369	Cyclosativene								0.05 ± 0.06
1373	1374	α-Copaene	2.24 ± 1.51	0.89 ± 0.14	0.83 ± 0.06	0.05 ± 0.08	0.04 ± 0.04	1.93 ± 0.43	1.36 ± 0.71	

1376	1374	Isoledene						0.02 ± 0.03	0.05 ± 0.09	
1386	1387	β-Cubebene	0.81 ± 0.23							
1388	1387	β-Bourbonene								0.02 ± 0.03
**1389**	**1389**	**β-Elemene**	1.07 ± 0.30	3.58 ± 0.27	2.83 ± 0.22	4.63 ± 1.21	4.91 ± 0.27	**9.92 ± 0.58**	**6.10 ± 1.32**	0.40 ± 0.05
1410	1409	α-Gurjunene	1.70 ± 0.76	0.64 ± 0.05	1.03 ± 0.20	0.05 ± 0.09		1.29 ± 0.31	1.37 ± 0.80	
1410	1411	*cis*-α-Bergamotene				0.02 ± 0.03				
1411	1410	α-Cedrene							0.96 ± 0.12	
**1417**	**1417**	**(*E*)-β-Caryophyllene**	2.06 ± 1.00	**5.72 ± 0.93**	3.04 ± 0.24	1.56 ± 0.27	2.07 ± 0.43	4.24 ± 0.70	2.04 ± 0.12	4.26 ± 1.32
1425	1432	*trans*-α-Bergamotene				0.71 ± 0.64				0.07 ± 0.05
1432	1430	β-Copaene	0.26 ± 0.09		0.08 ± 0.11					0.32 ± 0.10
**1433**	**1434**	**γ-Elemene**		0.17 ± 0.02	0.24 ± 0.06	**38.33 ± 3.80**	**44.36 ± 4.06**	0.34 ± 0.05	0.85 ± 0.83	0.22 ± 0.07
1435	1437	α-Guaiene			0.51 ± 0.11			0.57 ± 0.15	0.48 ± 0.23	
1443	1442	6,9-Guaiadiene				0.04 ± 0.07			0.03 ± 0.06	
1451	1452	α-Humulene	0.73 ± 0.18	1.15 ± 0.12	2.06 ± 0.07	0.43 ± 0.21	0.56 ± 0.12	2.53 ± 0.14	2.11 ± 0.67	
1453	1453	Geranyl acetone		0.22 ± 0.08	0.05 ± 0.06					0.05 ± 0.05
1458	1465	Thujopsadiene		0.38 ± 0.09						
1458	1458	*allo*-Aromadendrene							0.04 ± 0.07	
1458	1457	Sesquisabinene *								1.74 ± 0.71
1469	1475	γ-Gurjunene							0.12 ± 0.21	
1470	1471	Dauca-5,8-diene	0.91 ± 0.30	0.12 ± 0.07						
1482	1481	γ-Curcumene								0.03 ± 0.04
1485	1484	Germacrene D	1.20 ± 0.65	0.64 ± 0.26	2.03 ± 0.43	3.20 ± 1.36	3.41 ± 0.38	0.67 ± 0.40	0.52 ± 0.48	6.80 ± 1.52
**1485**	**1489**	**β-Selinene**	2.47 ± 0.68	2.00 ± 0.44	**6.88 ± 0.61**	0.32 ± 0.41	1.14 ± 0.33	**8.17 ± 0.57**	**7.54 ± 2.59**	
1488	1492	*cis*-β-Guaiene		0.55 ± 0.26		0.02 ± 0.03		0.07 ± 0.04	0.57 ± 0.11	
1489	1493	*trans*-Muurola-4(14),5-diene	0.76 ± 0.06							
1491	1493	*epi*-Cubebol	2.36 ± 3.33							

**1493**	**1496**	**Viridiflorene**			**7.65 ± 0.63**			**8.87 ± 0.84**	**12.42 ± 2.28**	
**1493**	**1500**	**Bicyclogermacrene**	**7.55 ± 4.95**	3.66 ± 0.31						
1496	1495	γ-Amorphene	0.10 ± 0.14					0.02 ± 0.03	0.94 ± 0.20	0.05 ± 0.04
1496	1500	α-Muurolene	1.07 ± 0.19							
1497	1493	α-Zingiberene								0.35 ± 0.07
**1501**	**1499**	**Curzerene**				**23.21 ± 4.96**	**23.39 ± 4.45**			
1503	1502	*trans*-β-Guaiene		0.18 ± 0.07	0.15 ± 0.21					
**1504**	**1505**	**β-Bisabolene**			1.23 ± 0.33					**5.84 ± 2.88**
1511	1511	δ-Amorphene	0.17 ± 0.24	1.12 ± 0.25	0.53 ± 0.06	0.04 ± 0.07			1.14 ± 0.46	
**1512**	**1514**	**Cubebol**	**6.49 ± 2.11**			0.10 ± 0.18		0.31 ± 0.14		
1513	1514	(*Z*)-γ-Bisabolene *					0.25 ± 0.07			
1517	1508	Germacrene A			0.63 ± 0.12	0.03 ± 0.05				
1518	1513	*trans*-Cycloisolongifol-5-ol							1.56 ± 1.49	
1522	1520	7-*epi*-α-Selinene			0.08 ± 0.11			0.11 ± 0.04		
**1522**	**1522**	**δ-Cadinene**	**6.04 ± 1.77**					0.24 ± 0.08	0.64 ± 1.11	
1525	1531	(*Z*)-Nerolidol *								0.82 ± 0.29
1526	1521	β-Sesquiphellandrene *								0.15 ± 0.16
1532	1532	γ-Cuprenene				0.10 ± 0.17				
1534	1529	(*E*)-γ-Bisabolene								0.12 ± 0.04
1536	1533	*trans*-Cadina-1,4-diene	0.59 ± 0.03	0.04 ± 0.04	0.13 ± 0.18			0.02 ± 0.03	0.09 ± 0.16	
1539	1539	α-Copaen-11-ol							0.03 ± 0.06	
1539	1540	(*E*)-α-Bisabolene (FFNSC)			0.53 ± 0.21					2.73 ± 1.41
**1547**	**1548**	**Elemol**	0.52 ± 0.02		**40.55 ± 4.87**	2.33 ± 0.57	0.89 ± 0.07	0.02 ± 0.03	0.54 ± 0.08	**49.78 ± 6.38**
1551	1554	β-Vetivenene							2.16 ± 1.06	
1561	1559	Germacrene B			0.30 ± 0.01	2.88 ± 2.56	1.21 ± 0.25			
1562	1561	(*E*)-Nerolidol	0.77 ± 1.08	3.51 ± 2.53	0.06 ± 0.08	0.98 ± 0.14	0.66 ± 0.15	4.82 ± 1.60	2.09 ± 1.22	0.30 ± 0.14

1562	1567	Palustrol	0.31 ± 0.00						0.07 ± 0.12	
1565	1562	*epi*-Longipinanol	0.20 ± 0.01							
1572	1574	Germacrene D-4-ol				0.03 ± 0.05		0.33 ± 0.16	0.14 ± 0.25	
**1574**	**1577**	**Spathulenol**	1.44 ± 0.07	**5.85 ± 0.48**		0.54 ± 0.14			0.31 ± 0.32	
1581	1582	Caryophyllene oxide	3.07 ± 0.67	0.80 ± 0.28	0.60 ± 0.12	0.05 ± 0.08			0.73 ± 0.32	
1588	1586	Thujopsan-2-α-ol			0.05 ± 0.07			0.08 ± 0.08		
1589	1592	Viridiflorol	0.75 ± 0.15							
1596	1596	Fokienol				0.95 ± 0.48		1.23 ± 0.23	2.91 ± 0.42	
1610	1602	Ledol							0.19 ± 0.33	
1611	1607	(*Z*)-Sesquilavandulol	0.31 ± 0.44							
1611	1608	β-Atlantol		2.13 ± 0.17		3.92 ± 1.50				
1618	1618	1,10-di-*epi*-Cubenol	2.05 ± 0.47	1.51 ± 0.28		0.30 ± 0.27			1.78 ± 0.50	
1618	1618	2,7*Z*-Bisaboladien-4-ol *					0.74 ± 0.27			
1622	1629	Eremoligenol				0.03 ± 0.06				
1625	1627	1-*epi*-Cubenol	1.59 ± 0.40							
**1626**	**1630**	**Muurola-4,10(14)-dien-1-β-ol**		**4.83 ± 0.64**		0.69 ± 0.14			3.05 ± 0.37	
1633	1635	*cis*-Cadin-4-en-7-ol						1.26 ± 0.06		
1638	1639	*allo*-Aromadendrene epoxide				0.03 ± 0.06			0.08 ± 0.13	
1639	1640	*epi*-α-Muurolol	5.22 ± 0.19							
1643	1645	Cubenol		0.77 ± 0.76		0.01 ± 0.02			0.24 ± 0.41	
**1644**	**1644**	**α-Muurolol**	**20.63 ± 2.81**							
1647	1649	β-Eudesmol			0.22 ± 0.05					0.03 ± 0.06
1649	1652	Himachalol	0.44 ± 0.08					0.14 ± 0.05		
1651	1652	α-Cadinol	1.53 ± 0.31							
1651	1651	Pogostol			0.82 ± 0.22	0.04 ± 0.07			1.51 ± 0.63	
1658	1652	α-Eudesmol								0.06 ± 0.10

1664	1666	14-Hydroxy-(*Z*)-caryophyllene *	0.25 ± 0.00							
1666	1668	14-hydroxy-9-*epi*-(*E*)-caryophyllene				0.02 ± 0.03			2.60 ± 1.16	
1675	1674	β-Bisabolol								0.02 ± 0.04
1679	1679	Khusinol		0.06 ± 0.11		0.05 ± 0.08			1.93 ± 0.69	
**1684**	**1685**	***α*-Bisabolol**			**17.97 ± 0.97**					**23.17 ± 1.28**
1686	1690	(*Z*)-*trans*-*α*-Bergamotol				0.19 ± 0.19				
1693	1700	Eudesm-7(11)-en-4-ol				0.04 ± 0.07				
1709	1713	(2*E*,6*Z*)-Farnesal	0.91 ± 0.83	0.24 ± 0.09	0.15 ± 0.21				0.04 ± 0.08	0.16 ± 0.20
1715	1714	(2*E*,6*Z*)-Farnesol	0.15 ± 0.21		0.15 ± 0.21					
1716	1713	14-Hydroxy-*α*-humulene							0.04 ± 0.08	
1722	1716	(*E*)-Nerolidyl acetate							0.03 ± 0.05	
1723	1722	(2*Z*,6*E*)-Farnesol								0.35 ± 0.22
1735	1733	Isobicyclogermacrenal		0.15 ± 0.06				0.06 ± 0.07		
1744	1740	(2*E*,6*E*)-Farnesal	1.61 ± 1.51	0.50 ± 0.17	0.36 ± 0.14				0.15 ± 0.27	0.32 ± 0.27
1758	1765	β-Costol				0.14 ± 0.24				
1910	1900	Dihydrocolumellarin				0.68 ± 0.23				
		**Monoterpene hydrocarbons**	0.00 ± 0.00	0.97 ± 0.47	0.00 ± 0.00	2.35 ± 1.87	0.00 ± 0.00	0.00 ± 0.00	0.00 ± 0.00	0.21 ± 0.03
		**Oxygenated monoterpenoids**	0.90 ± 0.30	6.97 ± 0.40	2.01 ± 0.22	0.11 ± 0.18	0.94 ± 0.17	0.28 ± 0.04	0.48 ± 0.24	0.69 ± 0.02
		**Sesquiterpene hydrocarbons**	34.62 ± 12.97	**58.48 ± 0.94**	33.14 ± 3.68	**59.35 ± 5.45**	**71.81 ± 4.47**	**90.90 ± 1.03**	**74.63 ± 2.38**	23.21 ± 8.20
		**Oxygenated sesquiterpenoids**	**50.55 ± 10.35**	20.36 ± 0.80	**60.90 ± 3.58**	34.33 ± 4.44	25.68 ± 4.30	8.25 ± 1.28	20.04 ± 0.76	**75.01 ± 7.89**
		**Others**	10.21 ± 1.36	11.46 ± 1.13	3.08 ± 0.29	2.79 ± 1.89	0.30 ± 0.19	0.08 ± 0.07	4.61 ± 1.71	0.21 ± 0.02
		**Total identified**	96.28 ± 0.95	98.23 ± 0.49	99.12 ± 0.40	98.93 ± 0.57	98.73 ± 0.70	99.51 ± 0.31	99.75 ± 0.43	99.33 ± 0.29

RI (L): Retention Index of Library; **RI (C):** Retention Index Calculated; **(*)** Tentative.

Table S2. Identification of chemical composition in the fruits of black pepper cultivars (Mean ± standard deviation - %).

RI (C)	RI (L)	Compound	Bragantina	Cingapura	Clonada	Equador	Guajarina	Uthirankota
768	762	1-Pentanol	0.40 ± 0.15	0.40 ± 0.14	0.26 ± 0.01	0.28 ± 0.03	0.09 ± 0.15	0.22 ± 0.01
788	788	1-Octene	0.61 ± 0.19	0.55 ± 0.23	0.35 ± 0.04	0.44 ± 0.03	0.12 ± 0.21	0.29 ± 0.03
819	815	(2*E*)-Octene	0.07 ± 0.07	0.05 ± 0.08				
920	921	Tricyclene		0.28 ± 0.06		0.02 ± 0.02	0.01 ± 0.02	0.03 ± 0.00
924	924	α-Thujene	0.48 ± 0.08		0.46 ± 0.02	0.95 ± 0.01	1.33 ± 0.26	1.21 ± 0.08
**931**	**932**	**α-Pinene**	**10.67 ± 0.82**	**10.51 ± 0.25**	**9.72 ± 0.28**	**8.21 ± 0.22**	**6.91 ± 0.49**	**9.71 ± 0.59**
946	946	Camphene					0.01 ± 0.02	
**967**	**969**	**Sabinene**	0.60 ± 1.04	0.01 ± 0.02	0.67 ± 1.16	**5.11 ± 0.14**	**8.82 ± 1.39**	1.80 ± 3.12
**976**	**974**	**β-Pinene**	**35.05 ± 2.44**	**27.10 ± 0.52**	**33.82 ± 1.26**	**26.93 ± 0.64**	**22.61 ± 2.76**	**29.82 ± 2.65**
988	988	Myrcene					2.24 ± 0.20	
999	1002	α-Phellandrene		1.42 ± 0.07	1.19 ± 0.07	0.71 ± 0.01	0.08 ± 0.14	
**1005**	**1008**	**δ-3-Carene**	0.02 ± 0.03	**11.41 ± 0.84**	**8.50 ± 0.58**	4.65 ± 0.24		
1016	1014	α-Terpinene					0.56 ± 0.63	0.53 ± 0.13
**1024**	**1024**	**Limonene**	**21.67 ± 1.77**	**30.12 ± 1.31**	**31.77 ± 0.61**	**27.95 ± 0.39**	**22.71 ± 4.50**	**21.00 ± 0.57**
1046	1044	(*E*)-β-Ocimene	0.02 ± 0.03				0.35 ± 0.04	0.06 ± 0.05
1052	1054	γ-Terpinene	0.35 ± 0.10	0.04 ± 0.04	0.16 ± 0.01	0.51 ± 0.01	1.17 ± 0.57	1.02 ± 0.25
1066	1065	*cis*-Sabinene Hydrate	0.67 ± 0.06		0.28 ± 0.02	1.46 ± 0.04	2.51 ± 0.57	1.54 ± 0.29
1084	1083	tetramethyl-Pyrazine	0.20 ± 0.02	0.08 ± 0.14	0.18 ± 0.15		0.20 ± 0.09	
1088	1086	Terpinolene	0.37 ± 0.06	0.91 ± 0.19	0.75 ± 0.16	0.52 ± 0.02	0.42 ± 0.20	0.40 ± 0.07
**1094**	**1095**	**Linalool**	1.70 ± 0.20	2.77 ± 0.40	2.19 ± 0.18	**6.01 ± 0.06**	4.08 ± 0.65	**5.05 ± 0.34**
1121	1118	*cis-p*-Menth-2-en-1-ol	0.24 ± 0.05		0.10 ± 0.01	0.31 ± 0.02	0.54 ± 0.29	0.55 ± 0.03
1133	1136	*trans-p*-Menth-2-en-1-ol	0.13 ± 0.03		0.01 ± 0.02	0.18 ± 0.01	0.23 ± 0.25	0.32 ± 0.01
1160	1166	*p*-Mentha-1,5-dien-8-ol		0.05 ± 0.01				
1167	1170	*cis*-Linalool Oxide	0.02 ± 0.03	0.32 ± 0.06				

**1174**	**1174**	**Terpinen-4-ol**	2.88 ± 0.48	0.20 ± 0.08	1.13 ± 0.06	3.67 ± 0.20	**8.10 ± 2.07**	**6.24 ± 0.35**
1178	1179	*p*-Cymen-8-ol	0.02 ± 0.03	0.24 ± 0.06	0.09 ± 0.01	0.13 ± 0.01		0.16 ± 0.01
1180	1183	Cryptone					0.09 ± 0.16	
1185	1186	α-Terpineol	1.13 ± 0.18	0.58 ± 0.04	0.84 ± 0.05	0.40 ± 0.02	1.04 ± 0.17	0.66 ± 0.03
1190	1195	*cis*-Piperitol	0.03 ± 0.03				0.06 ± 0.10	
1195	1194	Myrtenol		0.25 ± 0.02	0.10 ± 0.02	0.06 ± 0.01		
1202	1196	Safranal		0.07 ± 0.01				
1202	1207	*trans*-Piperitol					0.07 ± 0.12	0.10 ± 0.01
1208	1214	Linalyl formate		0.12 ± 0.01				
1213	1215	*trans*-Carveol	0.01 ± 0.02				0.01 ± 0.02	0.05 ± 0.01
1228	1227	Nerol	0.19 ± 0.03		0.07 ± 0.03	0.04 ± 0.07	0.10 ± 0.10	
1237	1239	Carvone	0.01 ± 0.01					0.03 ± 0.03
1245	1244	Car-3-en-2-one		0.04 ± 0.05				
1248	1249	Piperitone		0.01 ± 0.02				
1254	1257	Methyl citronellate						0.02 ± 0.02
1293	1293	2-Undecanone		0.02 ± 0.02	0.03 ± 0.01		0.14 ± 0.01	0.04 ± 0.00
1326	1329	Piperonal	0.21 ± 0.08	0.20 ± 0.02	0.04 ± 0.01	0.02 ± 0.03	0.16 ± 0.28	0.08 ± 0.01
1334	1335	δ-Elemene	0.06 ± 0.02	0.15 ± 0.05	0.01 ± 0.01	0.09 ± 0.02	0.11 ± 0.03	
1344	1346	α-Terpinyl acetate	0.15 ± 0.02					
1372	1374	α-Copaene	0.46 ± 0.12	0.42 ± 0.08			0.01 ± 0.01	
1386	1387	β-Cubebene	0.05 ± 0.04					
1388	1389	β-Elemene	0.14 ± 0.08	0.21 ± 0.05	0.35 ± 0.02	0.29 ± 0.05	0.27 ± 0.04	0.05 ± 0.00
1411	1411	*cis*-α-Bergamotene						0.25 ± 0.02
**1417**	**1417**	**(*E*)-β-Caryophyllene**	**5.74 ± 0.56**	**7.84 ± 0.46**	4.70 ± 0.23	**5.24 ± 0.32**	**6.35 ± 0.34**	2.22 ± 0.12
1425	1430	β-Copaene	0.01 ± 0.01					
1431	1432	*trans*-α-Bergamotene					0.02 ± 0.03	0.31 ± 0.03

1435	1437	α-Guaiene			0.02 ± 0.00			
1437	1434	γ-Elemene				0.85 ± 0.19	1.94 ± 0.66	
1451	1452	α-Humulene	0.39 ± 0.08	0.48 ± 0.07	0.40 ± 0.02	0.29 ± 0.04	0.39 ± 0.04	
1451	1454	(*E*)-β-Farnesene						0.36 ± 0.03
1465	1469	Dihydrosesquicineole						0.03 ± 0.02
1469	1471	Dauca-5,8-diene	0.01 ± 0.01					
1478	1484	Germacrene D	0.01 ± 0.02	0.01 ± 0.01		0.03 ± 0.03	0.02 ± 0.03	0.32 ± 0.03
1481	1483	*trans*-β-Bergamotene (FFNSC)						0.15 ± 0.01
1490	1489	β-Selinene	0.12 ± 0.03		0.45 ± 0.02	0.17 ± 0.04	0.39 ± 0.16	0.06 ± 0.00
1491	1493	*epi*-Cubebol	0.36 ± 0.13					
1491	1493	α-Zingiberene						0.31 ± 0.03
1492	1496	Viridiflorene			0.29 ± 0.02			
1496	1500	α-Muurolene	0.04 ± 0.02					
1500	1499	Curzerene				0.44 ± 0.13	1.69 ± 0.53	
1504	1500	Bicyclogermacrene					0.06 ± 0.11	
1504	1505	β-Bisabolene	0.44 ± 0.12	0.03 ± 0.02	0.14 ± 0.00	0.28 ± 0.04		0.54 ± 0.04
1511	1505	(*E*,*E*)-α-Farnesene *					0.04 ± 0.07	
1512	1514	Cubebol	0.52 ± 0.13	0.07 ± 0.02	0.03 ± 0.01	0.01 ± 0.02	0.02 ± 0.03	
1517	1508	Germacrene A						0.05 ± 0.01
1519	1511	δ-Amorphene	0.35 ± 0.08	0.19 ± 0.05				
1519	1521	β-Sesquiphellandrene						0.07 ± 0.01
1528	1529	(*E*)-γ-Bisabolene						0.30 ± 0.03
1539	1540	(*E*)-α-Bisabolene						0.05 ± 0.01
**1548**	**1548**	**Elemol**	0.02 ± 0.02	0.01 ± 0.01	0.10 ± 0.10	0.15 ± 0.02	0.26 ± 0.02	**7.01 ± 0.23**
1554	1559	Germacrene B					0.02 ± 0.03	
1559	1561	(*E*)-Nerolidol	0.01 ± 0.02	0.01 ± 0.02		0.83 ± 0.16	0.03 ± 0.05	0.01 ± 0.02

1575	1577	Spathulenol	0.08 ± 0.05	0.10 ± 0.06			0.07 ± 0.12	
1581	1582	Caryophyllene Oxide	0.52 ± 0.17	0.76 ± 0.17	0.49 ± 0.03	1.36 ± 0.20	1.69 ± 0.74	1.12 ± 0.07
1587	1596	Fokienol		0.13 ± 0.04		0.02 ± 0.02		
1606	1608	Humulene Epoxide II		0.02 ± 0.02	0.02 ± 0.00	0.11 ± 0.01	0.06 ± 0.10	0.02 ± 0.04
1608	1604	Khusimone						0.02 ± 0.04
1617	1608	β-Atlantol				0.02 ± 0.03	0.21 ± 0.19*	
1626	1629	Eremoligenol						0.13 ± 0.01
1628	1630	γ-Eudesmol						0.19 ± 0.04
1633	1630	Muurola-4,10(14)-dien-1β-ol		0.86 ± 0.18		0.62 ± 0.07	0.74 ± 0.37	
1633	1636	Gossonorol						0.14 ± 0.02
1638	1640	*epi*-α-Murrolol	0.24 ± 0.09					
1642	1639	*allo*-Aromadendrene epoxide					0.06 ± 0.11	0.01 ± 0.02
1643	1644	α-Muurolol	1.33 ± 0.45					
1643	1645	Cubenol					0.03 ± 0.05	
1648	1649	β-Eudesmol						0.49 ± 0.11
1651	1651	Pogostol			0.17 ± 0.04		0.06 ± 0.10	
1651	1652	α-Eudesmol						0.46 ± 0.07
1655	1656	α-Bisabolol Oxide B						0.04 ± 0.04
1665	1670	*epi*-β-Bisabolol						0.09 ± 0.01
1668	1668	14-Hydroxy-9-*epi*-(*E*)-caryophyllene				0.03 ± 0.03	0.07 ± 0.12	
1675	1679	Khusinol		0.11 ± 0.04				
1680	1685	Germacra-4(15),5,10(14)-trien-1α-ol					0.04 ± 0.06	
1683	1685	α-Bisabolol		0.02 ± 0.02				3.78 ± 0.26
1699	1700	Eudesm-7(11)-en-4-ol					0.01 ± 0.01	
1766	1773	α-Costol					0.15 ± 0.27	
1926	1929	Musk ambrette	0.07 ± 0.03	0.07 ± 0.04	0.02 ± 0.02			

1951	1959	Hexadecanoic Acid	0.01 ± 0.02					
2404	2400	Tetracosane	0.05 ± 0.09					
		**Monoterpene hydrocarbons**	**79.23 ± 3.43**	**81.80 ± 0.64**	**87.02 ± 0.72**	**75.77 ± 1.44**	**67.22 ± 6.69**	**65.59 ± 0.38**
		**Oxygenated monoterpenoids**	7.18 ± 1.02	4.54 ± 0.41	4.80 ± 0.27	12.26 ± 0.20	16.82 ± 4.45	14.71 ± 0.66
		**Sesquiterpenes hydrocarbons**	7.82 ± 1.08	9.32 ± 0.76	6.37 ± 0.31	7.24 ± 0.69	9.62 ± 0.55	5.04 ± 0.36
		**Oxygenated sesquiterpenoids**	3.08 ± 1.03	1.99 ± 0.50	0.81 ± 0.04	3.60 ± 0.64	5.19 ± 1.43	13.52 ± 0.48
		**Others**	1.63 ± 0.27	1.60 ± 0.56	0.88 ± 0.16	0.74 ± 0.03	0.71 ± 0.74	0.65 ± 0.06
		**Total identified**	98.93 ± 0.41	99.26 ± 0.13	99.88 ± 0.04	99.60 ± 0.23	99.56 ± 0.76	99.50 ± 0.06

RI (L): Retention Index of Library; **RI (C):** Retention Index Calculated; **(*)** Tentative.

Table S3. Docking energies (kJ/mol) for the essential oil ligands and their respective *Fusarium* target proteins.

**Ligand**	***F. oxysporum* cutinase**	***F. solani* cutinase**	***F. oxysporum* endoglucanase**	***F. oxysporum* feruloyl esterase**	***F. oxysporum* glucosamine-fructose-6-phosphate aminotransferase**	***F. odoratissimum* β-glucosidase**	***F. oxysporum* guanine nucleotide-binding protein subunit β**	***F. solani* ornithine decarboxylase**	***F. vanettenii* thiamine thiazole synthase**	***F. oxysporum* xylanase**
(*E*)-Anethole	-55.1	-51.9	-56.6	-43.5	-61.1	-62.2	-50.2	-69.8	-66.2	-59.7
Apiole	-66.2	-62.3	-75.1	-57.5	-77.7	-74.3	-70.6	-61.6	-73.5	-81.9
Bicyclogermacrene	-52.5	-58.9	-79.0	-59.6	-70.1	-66.8	-65.0	-69.9	-71.2	-73.0
β-Bisabolene	-68.7	-76.3	-87.0	-57.1	-79.3	-81.9	-74.2	-86.2	-93.0	-84.4
α-Bisabolol	-65.0	-75.1	-82.4	-59.4	-74.4	-88.6	-70.9	-69.5	-83.6	-87.2
δ-Cadinene	-54.9	-56.8	-68.2	-41.8	-57.6	-69.6	-58.5	-55.9	-70.8	-74.6
(*R*,*S*)-δ-3-Carene	-40.2	-45.2	-63.1	-53.0	-52.3	-59.6	-47.3	-61.7	-49.5	-54.0
(*S*,*R*)-δ-3-Carene	-41.4	-47.6	-65.1	-52.0	-55.2	-55.8	-44.4	-65.3	-49.5	-56.2
Carvacrol	-47.0	-49.6	-63.1	-40.9	-61.1	-64.0	-51.9	-67.5	-53.7	-59.5
(*E*)-β-Caryophyllene	-47.5	-52.6	-75.1	-62.7	-57.5	-61.9	-57.2	-62.2	-69.5	-58.3
Cubebol	-59.9	-61.2	-73.9	-57.9	-71.2	-72.3	-66.3	-74.2	-73.1	-80.1
Curzerene	-57.5	-62.5	-77.8	-68.0	-69.4	-75.9	-70.2	-69.6	-77.9	-80.9
Dillapiole	-58.8	-60.5	-72.3	-48.4	-76.8	-76.4	-64.5	-86.4	-71.0	-82.0
β-Elemene	-60.2	-58.5	-78.3	-59.1	-65.5	-71.9	-65.0	-56.7	-71.2	-73.6
δ-Elemene	-61.3	-58.6	-80.1	-55.7	-67.2	-75.4	-67.1	-70.3	-68.3	-74.6
γ-Elemene	-54.8	-55.8	-77.1	-57.3	-64.4	-64.2	-60.2	-69.6	-69.1	-72.7
α-Elemol	-65.1	-60.7	-81.9	-61.8	-66.8	-69.6	-66.8	-72.2	-75.2	-77.7
Eugenol	-57.0	-57.0	-70.2	-64.2	-69.0	-69.3	-61.1	-73.9	-77.9	-69.2
(*E*)-Isoosmorhizol	-60.1	-56.4	-63.3	-46.7	-67.3	-70.9	-56.6	-74.7	-66.5	-70.5
(*R*)-Limonene	-48.8	-49.7	-56.7	-38.7	-49.2	-61.4	-51.8	-65.8	-55.8	-58.1
(*S*)-Limonene	-44.4	-49.5	-57.3	-38.1	-53.5	-61.9	-50.1	-62.1	-53.9	-58.5
(*R*)-Linalool	-55.2	-56.1	-65.5	-59.1	-56.9	-66.7	-58.0	-56.7	-73.6	-64.4

(*S*)-Linalool	-55.9	-58.4	-67.0	-57.0	-61.6	-64.3	-57.9	-63.3	-70.8	-64.5
Methyl eugenol	-56.0	-56.0	-58.2	-46.6	-64.7	-71.6	-62.3	-77.4	-66.0	-69.0
Muurola-4,10(14)-dien-1β-ol	-54.2	-57.2	-74.3	-47.7	-68.7	-67.7	-62.9	-87.2	-69.1	-74.7
α-Muurolol	-54.5	-56.9	-69.1	-53.5	-69.8	-75.8	-58.0	-61.0	-69.3	-77.5
(*R*,*R*)-α-Pinene	-35.2	-37.8	-58.4	-49.6	-47.2	-46.4	-42.1	-47.7	-42.9	-48.1
(*S*,*S*)-α-Pinene	-35.1	-38.1	-57.8	-47.9	-48.0	-44.1	-40.9	-57.7	-41.9	-46.9
(*R*,*R*)-β-Pinene	-35.4	-38.0	-58.9	-49.8	-52.3	-45.1	-40.3	-37.3	-43.1	-47.6
(*S*,*S*)-β-Pinene	-35.7	-38.3	-58.0	-47.5	-49.0	-44.8	-41.4	-57.2	-42.6	-48.8
(+)-Sabinene	-46.2	-49.2	-63.7	-53.5	-55.5	-58.3	-49.7	-65.2	-50.8	-59.1
(-)-Sabinene	-52.6	-51.0	-65.4	-49.5	-56.4	-58.6	-48.3	-61.6	-50.5	-56.4
Safrole	-58.8	-56.9	-73.6	-46.5	-64.4	-75.5	-55.5	-74.5	-78.9	-67.4
β-Selinene	-56.5	-56.3	-69.5	-60.3	-67.4	-75.6	-62.3	-68.7	-69.6	-72.2
Spathulenol	-62.1	-59.0	-70.8	-43.6	-67.5	-69.6	-57.9	-73.1	-75.2	-79.1
(*R*)-Terpinen-4-ol	-47.9	-50.1	-64.4	-35.4	-56.5	-61.3	-48.2	-68.3	-63.4	-64.2
(*S*)-Terpinen-4-ol	-48.9	-50.3	-64.6	-41.2	-57.0	-60.2	-47.9	-68.5	-60.4	-64.7
Thymol	-48.4	-49.2	-61.3	-39.2	-52.3	-63.9	-50.3	-71.0	-61.6	-59.6
Viridiflorene	-60.6	-59.8	-77.7	-43.2	-69.8	-69.2	-52.7	-55.4	-70.3	-76.5
